# Complete Genome Sequence of a Plant-Derived Phenylpropanoid-Degrading Bacterium, Pseudomonas putida JYR-1

**DOI:** 10.1128/MRA.01152-19

**Published:** 2020-01-02

**Authors:** Yanshuo Han, Jian Tian, Yuzhong Li, Hor-Gil Hur, Dongfei Han

**Affiliations:** a Institute of Environment and Sustainable Development in Agriculture, Chinese Academy of Agricultural Sciences, Beijing, China; b College of Food Science and Technology, Agricultural University of Hebei, Baoding, Hebei Province, China; c Biotechnology Research Institute, Chinese Academy of Agricultural Sciences, Beijing, China; d School of Earth Sciences and Environmental Engineering, Gwangju Institute of Science and Technology (GIST), Gwangju, Republic of Korea; University of Arizona

## Abstract

Pseudomonas putida JYR-1 was isolated from soil contaminated with industrial oil because of its ability to utilize *trans*-anethole as a carbon and energy source. The complete genome is 5.41 Mb with 4,834 protein-coding genes. Study of this isolate will provide insight into biotransformation pathways for phenylpropanoids.

## ANNOUNCEMENT

Pseudomonas putida JYR-1 was isolated from soil contaminated with industrial oil in South Korea and is capable of degrading the plant-derived propylbenzene *trans*-anethole (1). Stanier’s basal minimal salt buffer (MSB) containing 0.5% (wt/vol) *trans*-anethole was used as a selective medium during isolation. Soil suspensions were incubated with rotary shaking at 200 rpm at 27°C (1). The ability of isolated strain JYR-1 to transform phenylpropanoids, including *trans*-anethole, isoeugenol, isosafrole, and *O*-methyl isoeugenol, to the corresponding aromatic aldehyde has been characterized by expression of the gene encoding *trans*-anethole oxygenase in Escherichia coli (2). Further kinetic assays revealed *trans*-anethole oxygenase to be a novel self-sufficient monooxygenase that catalyzes the complete process of oxygenation and cofactor cycling in one subunit (3). In addition, *p*-anisaldehyde dehydrogenase was characterized to convert aromatic benzaldehydes such as *p*-anisaldehyde, vanillin, veratraldehyde, and piperonal to corresponding aromatic acids, *p*-anisic acid, vanillic acid, veratric acid, and piperonylic acid (4). A gene cluster involved in phenylpropanoid biometabolism was previously identified from a 34-kb genome fragment from P. putida JYR-1, which gave JYR-1 the potential to be an industrial strain for producing fragrances from plant-derived chemicals (2, 4). Thus, we sequenced the genome of P. putida JYR-1 in this study.

A cell culture of P. putida JYR-1 was grown in LB broth at 27°C and stored with 30% glycerol at –80°C after isolation. Prior to genome sequencing, this stock culture was streaked on LB agar. A single colony was picked and cultured in LB broth followed by cell collection. Genomic DNA of JYR-1 was extracted with the SDS method (5). The quality of the extracted DNA was assessed using agarose gel electrophoresis and quantified fluorometrically using a Qubit fluorometer. Unamplified libraries were prepared with the SMRTbell template prep kit 1.0 using the Pacific Biosciences standard protocol. Genome sequencing was performed using a PacBio RS II platform (6). In total, 80,142 high-quality subreads were filtered using the stand-alone PRINSEQ-lite version (7) with an average read length of 7,694 bp (*N*_50_, 9,392 bp). *De novo* assembly was done using the Hierarchical Genome Assembly Process (HGAP) via SMRT Link 5.0.1 with two-round polishing using Quiver (8), generating a circular chromosome of 5,413,503 bp with an overall G+C content of 62.48%. Default parameters were used for all software unless otherwise specified.

The genome was annotated using the Kyoto Encyclopedia of Genes and Genomes (KEGG) (9) and Rapid Annotations using Subsystems Technology (RAST) 2.0 (10). We identified 4,834 protein-coding DNA sequences (CDSs) and 100 predicted RNAs (74 tRNAs, 22 rRNAs, and 4 noncoding RNAs [ncRNAs]). The public version of this genome in DDBJ/EMBL/GenBank (accession number CP043835) was annotated using the Prokaryotic Genome Annotation Pipeline (PGAP) (11). In this study, annotations from KEGG were used to determine functional categories, and RAST and PGAP combined with previous biochemical investigations (2, 4) were used for interpreting the function of individual genes.

From the functional annotation of KEGG, 782 CDSs in the category “metabolism of terpenoids and polyketides” and 64 CDSs in the category “xenobiotic biodegradation and metabolism” were characterized. In addition to the *trans*-anethole oxygenase gene (*tao*), the *p*-anisaldehyde dehydrogenase gene (*paad*), and the vanillate monooxygenase genes (*vanA*, *vanB*) reported in a previous biochemical study of Pseudomonas putida JYR-1 (2), many other genes involved in degradation of aromatic compounds were found in the JYR-1 genome. Various catalytic functions for phenylpropanoid metabolism predicted in the P. putida JYR-1 genome indicate its potential as a tool for biosynthesis of aromatic chemicals from plant-derived materials.

### Data availability.

The complete genome sequence of Pseudomonas putida JYR-1 has been deposited in DDBJ/EMBL/GenBank under the accession number CP043835. The raw reads were deposited in the Sequence Read Archive (SRA) under the accession number SRR10092016.

## References

[1] Ryu J, Seo J, Lee Y, Lim Y, Ahn JH, Hur HG 2005 Identification of *syn*- and *anti*-anethole-2,3-epoxides in the metabolism of *trans*-anethole by the newly isolated bacterium *Pseudomonas putida* JYR-1. J Agric Food Chem 53:5954–5958. doi:10.1021/jf040445x. 16028980

[2] Han D, Ryu JY, Kanaly RA, Hur HG 2012 Isolation of a gene responsible for the oxidation of *trans*-anethole to *para*-anisaldehyde by *Pseudomonas putida* JYR-1 and its expression in *Escherichia coli*. Appl Environ Microbiol 78:5238–5246. doi:10.1128/AEM.00781-12. 22610435 PMC3416440

[3] Han D, Sadowsky MJ, Chong Y, Hur HG 2013 Characterization of a self-sufficient *trans*-anethole oxygenase from *Pseudomonas putida* JYR-1. PLoS One 8:e73350. doi:10.1371/journal.pone.0073350. 24066043 PMC3774712

[4] Han D, Kurusarttra S, Ryu JY, Kanaly RA, Hur HG 2012 Production of natural fragrance aromatic acids by coexpression of *trans*-anethole oxygenase and *p*-anisaldehyde dehydrogenase genes of *Pseudomonas putida* JYR-1 in *Escherichia coli*. J Agric Food Chem 60:11972–11979. doi:10.1021/jf303531u. 23140548

[5] Sambrook J, Russell DW 2001 Molecular cloning: a laboratory manual, 3rd ed Cold Spring Harbor Laboratory Press, Cold Spring Harbor, NY.

[6] Ardui S, Ameur A, Vermeesch JR, Hestand MS 2018 Single molecule real-time (SMRT) sequencing comes of age: applications and utilities for medical diagnostics. Nucleic Acids Res 46:2159–2168. doi:10.1093/nar/gky066. 29401301 PMC5861413

[7] Schmieder R, Edwards R 2011 Quality control and preprocessing of metagenomic datasets. Bioinformatics 27:863–864. doi:10.1093/bioinformatics/btr026. 21278185 PMC3051327

[8] Chin CS, Alexander DH, Marks P, Klammer AA, Drake J, Heiner C, Clum A, Copeland A, Huddleston J, Eichler EE, Turner SW, Korlach J 2013 Nonhybrid, finished microbial genome assemblies from long-read SMRT sequencing data. Nat Methods 10:563–569. doi:10.1038/nmeth.2474. 23644548

[9] Kanehisa M, Sato Y, Kawashima M, Furumichi M, Tanabe M 2016 KEGG as a reference resource for gene and protein annotation. Nucleic Acids Res 44:D457–D462. doi:10.1093/nar/gkv1070. 26476454 PMC4702792

[10] Overbeek R, Olson R, Pusch GD, Olsen GJ, Davis JJ, Disz T, Edwards RA, Gerdes S, Parrello B, Shukla M, Vonstein V, Wattam AR, Xia F, Stevens R 2014 The SEED and the Rapid Annotation of microbial genomes using Subsystems Technology (RAST). Nucleic Acids Res 42:D206–D214. doi:10.1093/nar/gkt1226. 24293654 PMC3965101

[11] Tatusova T, DiCuccio M, Badretdin A, Chetvernin V, Nawrocki EP, Zaslavsky L, Lomsadze A, Pruitt KD, Borodovsky M, Ostell J 2016 NCBI Prokaryotic Genome Annotation Pipeline. Nucleic Acids Res 44:6614–6624. doi:10.1093/nar/gkw569. 27342282 PMC5001611

